# On-Chip 3D Printing of Polymer Waveguide-Coupled Single-Photon Emitter Based on Colloidal Quantum Dots

**DOI:** 10.3390/polym15092201

**Published:** 2023-05-06

**Authors:** Gia Long Ngo, Long Nguyen, Jean-Pierre Hermier, Ngoc Diep Lai

**Affiliations:** 1 LuMIn, ENS Paris-Saclay, CentraleSupélec, CNRS, Université Paris-Saclay, 91190 Gif-sur-Yvette, France; 2 GEMaC, UVSQ, CNRS, Université Paris-Saclay, 78000 Versailles, France; 3 Department of Physics and Astronomy, University of Rochester, Rochester, NY 14627, USA

**Keywords:** low-one photon absorption direct laser writing, single-photon emitters, quantum dots, polymeric photonic structures

## Abstract

In the field of quantum technology, there has been a growing interest in fully integrated systems that employ single photons due to their potential for high performance and scalability. Here, a simple method is demonstrated for creating on-chip 3D printed polymer waveguide-coupled single-photon emitters based on colloidal quantum dots (QDs). By using a simple low-one photon absorption technique, we were able to create a 3D polymeric crossed-arc waveguide structure with a bright QD on top. These waveguides can conduct both excitation laser and emitted single photons, which facilitates the characterization of single-photon signals at different outputs with a conventional confocal scanning system. To optimize the guiding effect of the polymeric waveguide structures, comprehensive 3D finite-difference time-domain simulations were performed. Our method provides a straightforward and cost-effective way to integrate high-performance single-photon sources with on-chip photonic devices, enabling scalable and versatile quantum photonic circuits for various applications.

## 1. Introduction

The key to future optical quantum computing lies in the development of high performance and scalable systems that can integrate different elements, such as single photon emitters (SPEs) and waveguides at the micro-scale level [1,2,3,4,5,6,7]. On-chip photonics are an attractive technology for the implementation of such systems. However, the efficient integration of SPEs with waveguide structures presents challenges due to the complexity of the fabrication processes and the low signal-to-noise ratio (SNR). In particular, to precisely place solid-state SPEs into waveguide structures, advanced and expensive techniques such as chemical vapor deposition [8,9], molecular beam epitaxial [10,11,12,13,14,15], electron-beam lithography [10,11,12,13,14,16,17], inductively coupled plasma etching [9,10,11,12,13,14,15,16,17], and two-photon lithography [16,18,19] have been commonly used. Meanwhile, the low signal-to-noise ratio is a problem related to the waveguide design and the combination of waveguide materials and SPEs [18].

Recently, our research group proposed a promising solution to address the above issues [20,21]. Specifically, we used a fine combination of the in situ low-one photon absorption (LOPA) direct laser writing (DLW) technique, SU-8 negative photoresist, and bright SPEs based on CdSe/CdS core-shell quantum dots (QDs). With this approach, we can deterministically integrate a QD-based SPE into arbitrary polymeric structures. The excitation wavelength of 532 nm (continuous-wave laser) used in the LOPA-DLW method has two key advantages. Firstly, it facilitates the fabrication of 3D polymeric structures [22]. Secondly, it cancels the blinking effect of CdSe/CdS QDs, resulting in stable quantum devices [23,24]. At room temperature, the SNR of CdSe/CdS QDs is about 30 at a maximum single-photon count rate of $1.5\times 10^{6}$ counts per second (cps) [20]. To put this into perspective, our QDs are approximately ten times brighter than the SPEs based on nitrogen-vacancy (NV) color centers in diamond used in other studies [18,19]. Moreover, the QDs can be excited with an optical power of only a few μW compared to about 1 mW for NV centers, which significantly reduces the background signal.

In this work, we use the simple and low-cost LOPA-DLW setup to manufacture a 3D polymeric crossed-arc waveguide structure featuring a bright single QD at the top. The idea for the vertical arc shape structure comes from the fact that most research on waveguide-coupled SPE relies on planar configurations using semiconductor materials. The vertical structure has advantages such as simpler incoupling and outcoupling and eliminates the need for fabricating gratings for signal coupling, which is required in planar waveguides. Additionally, the low refractive index of the polymer performs better in the vertical configuration. The basic concept is that the fabricated waveguides simultaneously guide both the excitation laser and the emitted single-photon signal, enabling the characterization of a single-photon signal at all four outputs (legs of the waveguide) using a conventional confocal system. Our approach differs from most other waveguide-coupled SPEs that require two separate systems for excitation and signal acquisition at the waveguide output. To enhance the guiding efficiency of the polymeric waveguide structures, we conducted detailed 3D finite-difference time-domain (FDTD) simulations to identify the optimal waveguide size. Our method provides a convenient and low-cost solution for integrating high performance single-photon sources with on-chip photonic devices. In particular, the on-chip 3D printing technique used in this work can be easily adapted to different material systems and waveguide designs, opening up new possibilities for the development of scalable integrated quantum photonics.

## 2. Methods

### 2.1. Experiment Setup

**Figure 1:**
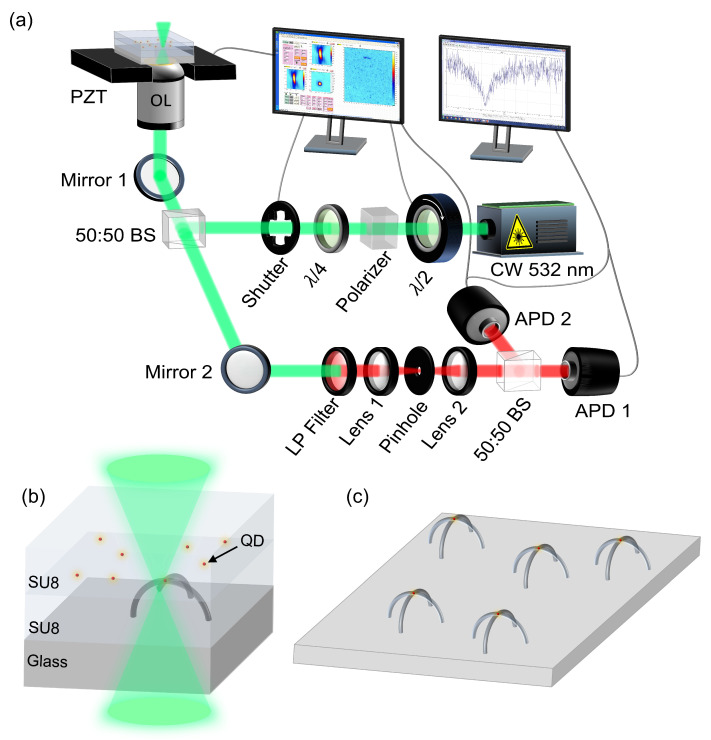
(**a**) LOPA-DLW setup. (**b**,**c**) Illustrations of sample preparation of polymeric crossed-arc waveguide containing a single QD using the LOPA-DLW technique.

Our study is based on a LOPA-DLW configuration that integrates a confocal scanning microscope with a Hanbury Brown and Twiss (HBT) experiment (Figure 1a). Specifically, the system incorporates a 532 nm continuous-wave laser, of which power can be fine tuned via a polarizer and a half-wave plate mounted on an electronic rotator. The laser beam is tightly focused onto the sample using an oil immersion objective lens with a numerical aperture (NA) of 1.3. To achieve a circular focusing spot, a quarter-wave plate modifies the polarization state of the excitation light to circular polarization before being focused. With the use of multiple optical instruments, the final laser power can be varied from 0 to 50 mW. We placed the sample on a 3D piezoelectric translation stage (PZT) with a maximum displacement of 300 μm and a resolution of 0.1 nm. The exposure time is controlled by an electronic shutter that can switch in as little as 2.5 μs. We utilized a long-pass filter with a cutoff wavelength of 580 nm to eliminate all residual excitation green light. Finally, the signal was sent to the HBT system to test for single-photon purity, and the count rate from one of the two detectors was used to generate 2D mapping images of the sample. All the equipment was synchronized and regulated using a self-developed LabVIEW program, which allowed for seamless integration of the various components of the LOPA-DLW setup.

### 2.2. Sample Preparation

A negative photoresist (SU-8 2005, MicroChem Corp., Westborough, MA, USA) was used to create a polymer film with a thickness of 5 μm. This was achieved by spin-coating the photoresist onto a 150 μm thick clean glass slide at a rotation speed of 3000 rpm, followed by a soft bake process of 1 min at 65 ${}^{{}^{\circ}}$C and 3 min at 95 ${}^{{}^{\circ}}$C. Next, 30 μL of solution containing colloidal CdSe/CdS QDs was spin-coated on top of the polymer film to create a horizontal layer of randomly distributed QDs. We note that the QDs solution was first diluted $10^{5}$ times from a stock solution of 3 μM. Another layer of SU-8 photoresist was then applied on top, resulting in the distribution of the QDs particles in a plane parallel to the glass/SU-8 interface and completely inside the SU-8 material. The colloidal QDs were synthesized through the chemical method and had a spherical shape with an average diameter of approximately 13 nm, as described in Ref. [25].

To fabricate vertical crossed-arc waveguide structures containing a single QD on top, the sample was placed into the LOPA-DLW setup. In the first step, a low laser power of 5 μW is used to scan the sample in both the xy and xz planes and precisely determine the *x*, *y*, and *z* coordinates of the QDs randomly dispersed within the polymer film. This power is sufficient to detect QDs with ease and does not have any impact on the polymer material. To confirm that a QD is a SPE, the sample is tested using the HBT system. Once a QD is confirmed as an SPE, the laser power is increased to 7 mW to initiate the fabrication of 3D waveguides containing SPE inside. Figure 1b illustrates this process. Finally, the sample was developed in SU-8 developer for 10 min to remove the unexposed photoresist and to obtain a solid structure (Figure 1c).

### 2.3. Numerical Calculation Method

To gain a better understanding of how single-photon signals and laser excitations propagate inside polymer waveguides and optimize the structure size for the guiding effect, we performed numerical simulations using the Ansys Lumerical software, which is a commercial 3D FDTD solver.

The simulated crossed-arc structure includes two arcs intersecting at the vertex. They lie within two vertical planes perpendicular to each other, with each arc having a circular cross-section maintained throughout its length. The radius of the arc (*R*) is kept fixed by *R* = 5 μm. We repeat that the standard thickness of commercial polymer SU-8 2005 after spincoating onto a glass surface is 5 μm. Therefore, we can easily work with *R* = 5 μm for this study, which serves as a proof of concept for 3D waveguide. Further studies regarding scalable systems will explore different *R* values. The refractive indices of the materials, such as glass, polymer, and air, were set to their respective values at the visible range (1.52, 1.6, and 1, respectively). In order to replicate the excitation laser beam in the LOPA-DLW setup, we computed the simulations with a circularly polarized Gaussian beam, characterized by a wavelength of 532 nm and a NA of 1.3. For the SPE, we used a model for a spherical QD represented by an incoherent sum of two orthogonal electric dipoles known as 2D transition dipoles with an emission wavelength of 620 nm. The QD’s orientation in space is determined by its *c*-axis (perpendicular to the plane containing the two dipoles), which forms an angle δ with the *z*-axis. The orientation of the *c*-axis can possibly be out-of-plane (OP) when $\delta =0^{{}^{\circ}}$ or in-plane (IP) when $\delta =90^{{}^{\circ}}$. The QD is located at the center of the cross-section at the top of the two arc waveguides, i.e., entirely inside the polymer material.

We ensured the reliability of our simulations by performing convergence tests on various simulation parameters, such as the size of the simulation region, grid sizes, simulation time, and boundary conditions. We specifically used an “override mesh region” with a grid divided equally at a 10 nm resolution in all *x*, *y*, and *z* directions on the entire crossed-arc structure.

## 3. Results and Discussion

### 3.1. Numerical Results

#### 3.1.1. Guiding the Emission of a Single Dipole

**Figure 2:**
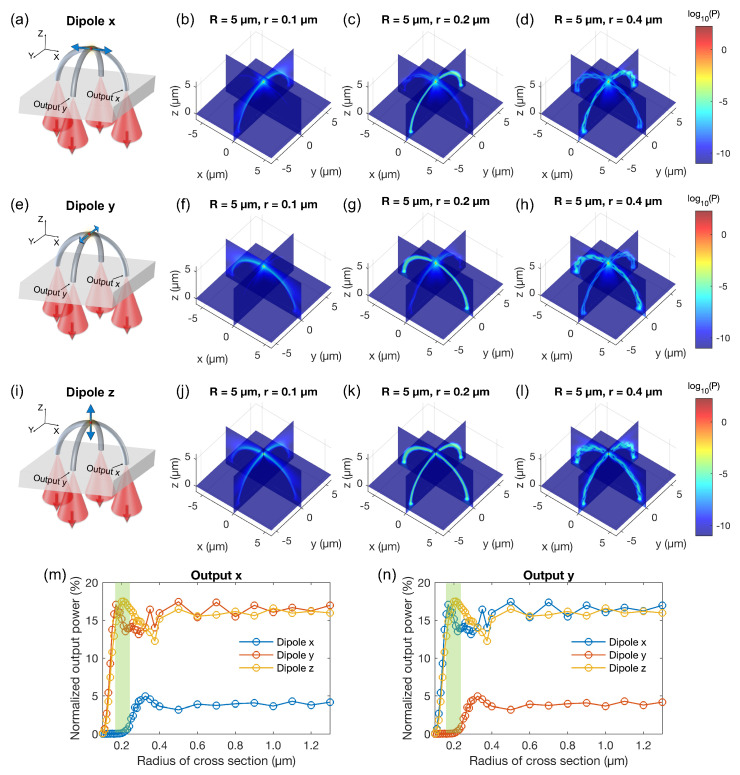
Numerical results for the propagation of the emission signal from a single electric dipole within the cross-arc waveguide structure. (**a**) Scheme for a *x*-oriented dipole located on top of an arc waveguide. The emitted signal from the dipole source is routed down to four outputs. Due to the symmetry, two outputs of the same arc are equivalent; here, only output *x* and output *y* are denoted. (**b**–**d**) Calculated Poynting vector magnitude distributions for the case depicted in (**a**) with the radius of the cross-section of the arc waveguide being 0.1, 0.2, and 0.4 μm, respectively. Results obtained from three monitors located in the xy, xz, and yz planes in the FDTD simulation region are combined for better visualization. (**e**–**h**) are similar to (**a**–**d**) but for a *y*-oriented dipole. (**i**–**l**) are similar to (**a**–**d**) but for a *z*-oriented dipole. (**m**,**n**) Normalized output power as a function of the cross-section radius for output *x* and output *y*, respectively. Normalized output power is calculated as the energy passing through the cross-section of the waveguide at the grass/air interface divided by the total energy emitted by the electric dipole throughout the space in all directions.

Section 2.3 introduces the two-dipoles model for a spherical QD. Our approach involves calculating the single dipole case first and then aggregating the results to obtain the incoherence sum of the two dipoles. The propagation of the emission signal from a single electric dipole within the cross-arc waveguide structure is shown in Figure 2. Illustrative models for *x*-, *y*-, and *z*-oriented dipoles positioned on top of an arc waveguide are displayed in Figure 2a, Figure 2e, and Figure 2i, respectively. Due to symmetry, two outputs at the base of an arc are identical. Therefore, only output *x* and output *y* are designated. The computed Poynting vector magnitude distributions reveal that the signal will be transmitted more effectively to output *y* when the dipole is oriented in the *x*-axis (Figure 2b–d) and vice versa (Figure 2f–h); when the dipole is oriented in the *z*-axis, the output signal at both outputs is the same (Figure 2j–l). This is a result of the emission properties of an electric dipole as an omnidirectional antenna, radiating equal power in all directions perpendicular to its axis, with power decreasing to zero on the axis. To improve visualization, we combined results from three monitors in the FDTD simulation region in three planes xy, xz, and yz, and $\log_{10}\left(P\right)$ is plotted instead of *P*.

We vary the radius of the circular cross-section (*r*) of the arc waveguide to determine the value that corresponds to the best guiding efficiency for fabrication. Poynting vector magnitude distributions are shown for three typical radii of 0.1 μm (large loss when the waveguide is too small), 0.2 μm (single-mode waveguide), and 0.4 μm (multi-mode waveguide). Additionally, the normalized output power as a function of the cross-section radius for output *x* and output *y* is presented in Figure 2m and Figure 2n, respectively. Normalized output power is calculated as the energy passing through the cross-section of the waveguide at the grass/air interface divided by the total energy emitted by the electric dipole throughout the space in all directions. It can be observed that the waveguide radius is the critical parameter for both the emitter-waveguide coupling and guiding losses. We choose to operate in single mode configuration with a waveguide radius of around 0.2 μm, as larger waveguide radius lead to multi-mode waveguides and lower collection efficiency. In particular, for a radius of 0.2 μm, approximately 17 % of the energy emitted by dipoles *y* and *z* will be transmitted to output *x*, while this percentage is almost negligible for dipole *x* (but is around 5% for the multi-mode configuration, this highlights the trade-off with respect to the collection efficiency).

#### 3.1.2. Guiding the Emission of Two Orthogonal Dipoles

**Figure 3:**
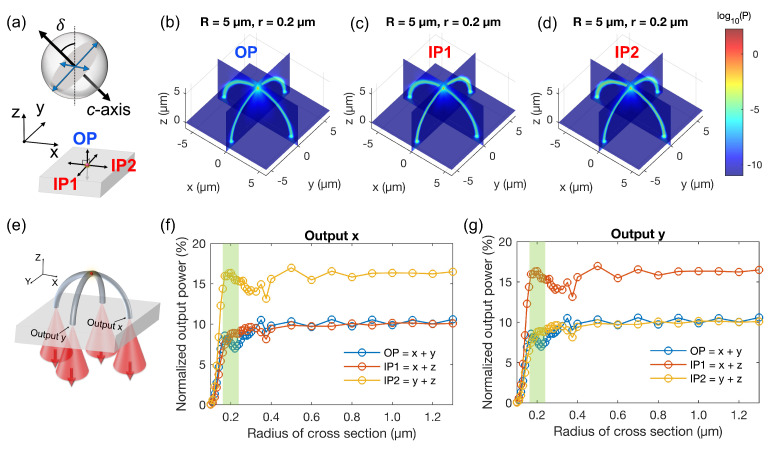
Numerical results for the propagation of the emission signal from two orthogonal electric dipoles within the crossed-arc waveguide structure. (**a**) Scheme for a QD-based SPE represented by an incoherent sum of two orthogonal electric dipoles. (**b**–**d**) Calculated Poynting vector magnitude distributions for the case depicted in (**e**) with three different orientations of the *c*-axis. In all three figures, r=0.2 μm. (**e**) Scheme for a QD-based SPE located on top of an arc waveguide. (**f**,**g**) Normalized output power as a function of the cross-section radius for output *x* and output *y*, respectively.

After obtaining the calculation results for the single-dipole cases, it was straightforward to combine them and obtain the results for the two-dipoles model. The Poynting vector magnitude distributions for the three different orientations of the *c*-axis, namely OP, IP1, and IP2 (Figure 3a), are presented in Figure 3b–d for the same waveguide radius of 0.2 μm. Here, OP refers to the sum of dipole *x* and dipole *y*, IP1 refers to the sum of dipole *x* and dipole *z*, and IP2 refers to the sum of dipole *y* and dipole *z*.

It can be observed that when the results for two orthogonal dipoles are plotted instead of one, the signal from the QD is transmitted to all four outputs, although there is still a preferred direction. For a waveguide radius of 0.2 μm, about 10% of the energy emitted by QD with OP and IP1 *c*-axis will be directed to output *x*, while this figure is 15% with IP2 *c*-axis, as shown in Figure 3f. For output *y*, IP1 and IP2 switch roles (Figure 3g). These results demonstrate that the crossed-arc waveguide structure acts as an optical splitter, enabling the signal emitted from the QD-based SPE to be split to all four outputs.

Now, there is another important issue that needs to be addressed, namely how to excite the SPE inside the 3D waveguide structure. Further investigation is required to determine the method for exciting the SPE and maximizing the efficiency of the optical splitter.

#### 3.1.3. Laser Excitation Configuration

**Figure 4:**
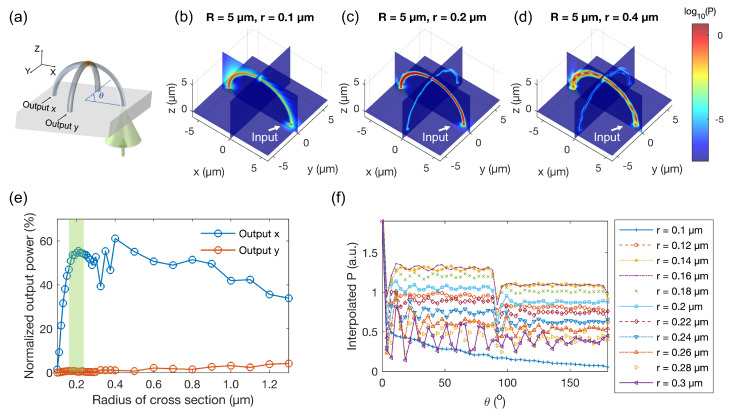
Numerical results for the propagation of the excitation laser within the crossed-arc waveguide structure. (**a**) Scheme for the case of focusing a Gaussian beam to the leg of an arc waveguide. (**b**–**d**) Calculated Poynting vector magnitude distributions for the case depicted in (**a**) with the radius of the cross-section of the arc waveguide being 0.1, 0.2, and 0.4 μm, respectively. (**e**) Normalized output power as a function of the cross-section radius. (**f**) Calculated Poynting vector magnitude distributions along the center of the cross-section of the excited arc. θ is denoted in (**a**).

Our proposal is to utilize a sole LOPA-DLW system with a single green laser for two objectives: fabricating the structures and characterizing single-photon signals at the outputs. The challenge is to excite and capture the signal simultaneously using the same objective lens. To simulate the excitation process, we focus a circularly polarized Gaussian beam with a wavelength of 532 nm and an NA of 1.3 (equivalent to the objective lens used in the experiment) onto one leg of the arc waveguide structure. Figure 4a depicts the scheme. Output *x* and output *y* are also labeled similarly to previous simulations. The calculated Poynting vector magnitude distributions with the waveguide radii being 0.1 μm (large loss), 0.2 μm (single-mode), and 0.4 μm (multi-mode) are presented in Figure 4b–d, respectively. The results show that a waveguide with r=0.2 μm also acts as a single-mode waveguide for the excitation laser signal. Moreover, most of the excitation light will be directed primarily to output *x*. That is, light tends to continue straight when it meets the intersection at the top of the crossed arc. With r=0.2 μm, approximately 55% of the excitation energy is directed to output *x*, while this figure is substantially smaller at output y (Figure 4e).

It should be noted that the size of QDs is only about 13 nm, which is much smaller than the waveguide diameter. To more precisely evaluate the excitation effect, we interpolated the Poynting vector along a curve centered on the excited arc waveguide (Figure 4f). At the position of the QD, which corresponds to $\theta =90^{{}^{\circ}}$ (θ is denoted in Figure 4a), there is a drop in the Poynting vector magnitude since this is where the two arcs meet. With a radius of 0.2 μm, the Poynting vector magnitude at $90^{{}^{\circ}}$ is approximately 30% of the value at $0^{{}^{\circ}}$ (when directly exciting a QD located at the glass/air interface). Ultimately, we found that the optimal excitation configuration is achieved with a radius range of 0.14–0.2 μm.

### 3.2. Experimental Results

**Figure 5:**
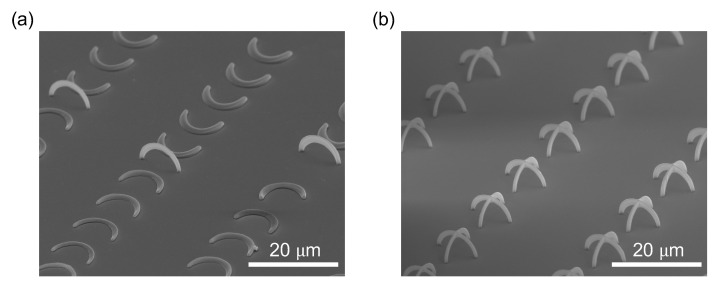
Comparison between the fabrication of (**a**) single-arc waveguide and (**b**) cross-arc waveguide.

The calculation results have demonstrated that the optimal waveguide radius for excitation and emission guiding effects is approximately 0.2 μm. To achieve such structure, we conducted experiments with various fabrication parameters. Figure 5 shows scanning electron microscope (SEM) images of two sets of fabricated structures, single-arc waveguides and crossed-arc waveguides, created using the same fabrication parameters. It is evident from the SEM images that the single-arc structure is highly susceptible to collapsing during sample development, whereas the crossed-arc structure exhibits high stability. Therefore, despite the simulation results indicating that the single-arc design may perform similarly to the crossed-arc design, even in terms of the excitation, it would be preferable to avoid any drop in the Poynting vector magnitude at $\theta =90^{{}^{\circ}}$; we do not use the former due to its instability. Although it is difficult to quantify the mechanical strength of the polymer structure, the fact that collapsed single arc waveguides were able to maintain their shape indicates that the mechanical strength of the 3D polymer structure produced using our method is sufficient for larger size applications, making it a scalable solution.

**Figure 6:**
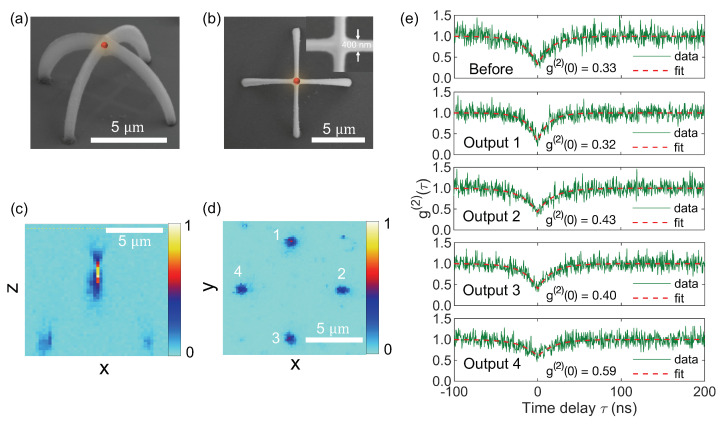
Experimental characterization results. SEM images (**a**) side view and (**b**) top view of the crossed-arc waveguide structure. Confocal scanning images in (**c**) xz plane and (**d**) xy plane of the crossed-arc waveguide structure containing a QD-based SPE on top. (**e**) Antibunching curves of a QD in polymeric film before fabricating the structure (upper) and of the guided signals at the four outputs at the legs of fabricated crossed-arc structure (lower). This result is measured by the same confocal system used for fabrication (excitation and acquisition through one objective lens simultaneously).

Figure 6 illustrates the experimental results of characterizing a cross-arc waveguide structure containing a QD located on top. The SEM images in Figure 6a,b show the side and top views of the fabricated crossed-arc structure, respectively. Since the QD’s dimensions are only about 13 nm and are entirely inside the polymer structure, we have intentionally added a red dot to indicate its relative position. As explained in Section 2.2, the QDs are initially placed in a sandwich structure between two polymer layers. During direct laser writing, when the focusing laser spot passes through the exact location of the predefined QD, it is expected to be situated in the center of the waveguide cross-section, rather than at the true top and exposed to air. Due to inherent asymmetric laser spot and limitations in the fabrication process, it was not possible to achieve uniform waveguide radius. As a result, variations were observed in the radius values along different directions. Specifically, based on the analysis of electron micrographs, the radius of the fabricated waveguide was found to be 0.2 μm in the xy plane and 0.5 μm in the *z*-axis. We note that while the experimental structure does not differ significantly from the simulated one, it is challenging in practice to precisely simulate the experimental structure due to factors such as surface roughness, uneven deformation along the cross-section of the waveguide, positional errors of the QD within the waveguide, and the need to repeat convergence tests for all these simulations.

The presence of a luminous QD situated atop the structure is validated by the confocal scanning image captured in the xz plane (Figure 6c). Meanwhile, the confocal scanning image in the xy plane (glass/air interface) shows four spots equivalent to four outputs named from 1 to 4. The reason for the central QD being absent in the xy scanning image is that conventional confocal scanning microscope systems are designed to detect signals only within the volumetric region of the laser focal spot. In our study, using an oil-immersion objective lens with an NA of 1.3, the size of the focusing spot along the *z*-axis is only about 700 nm, which is much smaller than the 5 μm distance from the QD located on top of the crossed-arc waveguide structure to the glass surface. Antibunching curves of a QD in polymeric film before fabricating the structure (upper) and of the guided signals at the four outputs were measured using the HBT setup at the same excitation laser power of 5 μW (Figure 6e). Here, $g^{\left(2\right)}\left(0\right)$ refers to the second-order correlation function measured at zero time delay between two detectors. It is a measure of the probability of detecting two photons at the same time in the two detectors. In experiments, a value of $g^{\left(2\right)}\left(0\right)$ < 0.5 is commonly accepted as an indication of single-photon emission. It is observed that, except for output 4 with $g^{\left(2\right)}\left(0\right)$ > 0.5, the remaining three outputs have a clear single photon signal with $g^{\left(2\right)}\left(0\right)$ < 0.5. Noise in antibunching curves can arise from various sources, such as unwanted signals emitted by the substrate and waveguide material, dark counts of photodiodes, or other sources of noise. Outputs 1 and 3 exhibit $g^{\left(2\right)}\left(0\right)$ smaller than the other two, suggesting that the QD in this case has a *c*-axis closer to IP1 than OP and IP2 (Figure 3g). In addition, imperfections in the waveguide structure may also contribute to the disparity between the signals received at the four outputs.

Our findings indicate that the 3D waveguides produced by the LOPA-DLW technique are proficient in guiding both the excitation laser and single photon emission. Additionally, the use of a straightforward confocal system for both excitation and signal capture implies the existence of a virtual SPE positioned at the output location. As a result, the combination of multiple crossed-arc waveguide structures holds promise for generating flawless arrays of SPE, which is a major objective for numerous investigations in this field [26,27,28,29,30,31,32].

## 4. Conclusions

In summary, we have demonstrated a low-cost and simple method for creating an on-chip 3D printed waveguide-coupled SPE based on colloidal QDs. Our approach involved using a continuous-wave laser with a low absorption wavelength for the polymer material to fabricate a 3D polymeric crossed-arc waveguide structure featuring a bright QD on top. The fabricated waveguides can conduct both the excitation laser and emitted single photons, allowing for the characterization of single-photon signals at the outputs using the same LOPA-DLW setup.

FDTD simulation results indicate that the waveguide radius is a crucial factor for both emitter-waveguide coupling and guiding losses. We chose to operate in a single-mode configuration with a waveguide radius of around 0.2 μm, based on calculations using a two-dipoles model to describe a QD. If the waveguide radius is too large, it will lead to multi-mode operation and lower collection efficiency. Fortunately, the radius of 0.2 μm is also nearly optimal for the excitation configuration.

Although the experimental structure slightly differs from the optimum simulation parameters, the experimental results demonstrate that the single-photon signal appears at three out of four outputs, indicating the effectiveness of the 3D waveguides in guiding both the excitation signal and the single-photon emission. Additionally, the ability to use a simple confocal system to capture the output signal suggests the existence of an imaginary SPE at the output location. This observation shows that our method holds great potential for creating perfect arrays of SPE by integrating multiple crossed-arc waveguide structures.

The results of this study are promising and provide a foundation for future work in the development of fully integrated on-chip single-photon sources for quantum technology.

## Data Availability

The data that support the findings of this study are available from the corresponding author upon reasonable request.
